# Sex-Specific Effect of Juvenile Diet on Adult Disease Resistance in a Field Cricket

**DOI:** 10.1371/journal.pone.0061301

**Published:** 2013-04-18

**Authors:** Clint D. Kelly, Brittany R. Tawes

**Affiliations:** Department of Ecology, Evolution & Organismal Biology, Iowa State University, Ames, Iowa, United States of America; CNRS, Université de Bourgogne, France

## Abstract

Food limitation is expected to reduce an individual’s body condition (body mass scaled to body size) and cause a trade-off between growth and other fitness-related traits, such as immunity. We tested the condition-dependence of growth and disease resistance in male and female *Gryllus texensis* field crickets by manipulating diet quality via nutrient content for their entire life and then subjecting individuals to a host resistance test using the live bacterium *Serratia marcescens*. As predicted, crickets on a high-quality diet eclosed more quickly, and at a larger body size and mass. Crickets on a high-quality diet were not in better condition at the time of eclosion, but they were in better condition 7–11 days after eclosion, with females also being in better condition than males. Despite being in better condition, however, females provided with a high-quality diet had significantly poorer disease resistance than females on a low-quality diet and in poor condition. Similarly, males on low- and high-quality diets did not differ in their disease resistance, despite differing in their body condition. A sex difference in disease resistance under diet-restriction suggests that females might allocate resources toward immunity during development if they expect harsh environmental conditions as an adult or it might suggest that females allocate resources toward other life history activities (i.e. reproduction) when food availability increases. We do not know what immune effectors were altered under diet-restriction to increase disease resistance, but our findings suggest that increased immune function might provide an explanation for the sexually-dimorphic increase in longevity generally observed in diet-restricted animals.

## Introduction

The resource pool from which individuals allocate to competing fitness-related life history traits is known as condition [1]. Individuals vary in condition because of genetic differences in acquisition and assimilation ability as well as resource availability in the environment [1], [2]. That adult life history decisions are contingent upon the resources accumulated during the juvenile life stage means that the environment experienced during early growth and development can have permanent effects on the adult phenotype and its subsequent performance [3]–[5]. This is particularly true in animals having several life stages, [6]–[8] such as insects [7], [9]. For example in stressful environments, such as when food quality or quantity is limited, insects tend to mature at a smaller body size, at an older age, and in poorer condition because rates of growth and development are diminished [9], [10]. This might prove costly to fitness if smaller adults in poorer condition suffer from decreased reproductive success and increased mortality [9]–[11].

The immune defense system is undoubtedly critical to fitness in animals as the ability to combat infection and disease increases survival [12]. However, because immunological responses are costly and condition-dependent [13]–[21], they should be subject to the same resource-allocation constraints as other fitness-related life history traits, such as growth and development [12], [22], [23]. Indeed, there is mounting evidence in insects that individuals experiencing nutrient-deficient environments during the larval or nymphal life stage have poor immunity as adults. For example, both starvation and nutrient deficiency during the larval stage increase susceptibility to viral infection in adult *Aedes aegytpi* mosquitoes through down-regulation of genes coding for antimicrobial peptides [24]. Reducing the amount of food given to larvae of the damselfly *Lestes viridis,* significantly decreases hemocyte number (circulating immune-competent cells) [25], phenoloxidase [25], [26] and pro-phenoloxidase [27] activities in adults. *Anopheles gambiae* mosquitoes provisioned with less food as larvae also exhibit a diminished capacity to melanize foreign particles [28].

The mechanism underlying the impact of nutritional conditions during ontogeny on adult immunity remains elusive. One possibility is that increased quality or quantity of food could simply improve overall body condition in a non-specific way, resulting in superior performance of all fitness-related traits, including immune defence [29]. Indeed, reducing larval food quality or availability can not only reduce adult immune function, but also produce smaller adults [24], [28], which in the case of damselflies, can also have diminished fat reserves [25].

Another possibility is that food availability during ontogeny could influence the proportion of resources allocated to immunity independently of its impact on body condition [29]. This could perhaps arise if greater food availability meant that a limiting and immune-specific metabolic resource is more readily available, or from increased resource allocation to the growth and development of tissues having immunological function [29], [30]. This possibility is supported by Fellous and Lazarro’s [29] findings that protein-deficient larval *D. melanogaster* had reduced transcription of immune-related genes independent of body condition as adults. Autumnal moths (*Epirrita autumnat*a) reared as larvae on low-quality diets had greater encapsulation ability as pupae, but not less body mass, than those on high-quality diets, suggesting that a nutrient was missing from the low-quality diet that affected immunity but not general body condition [31].

Investment in immunity is often sex-specific with females typically having superior immunity than males [22], [32], [33]. This pattern of investment is assumed to reflect how each sex maximizes fitness [22], [34] with investment in immunity depending upon the relationship between condition and reproduction and the extent to which parasites impact condition [35]. If parasitic impact and condition-dependence of reproduction is greater in females, then allocation to immunity should be higher in females [35]. Otherwise, if sexual selection is more condition-dependent in males, then males should protect their condition by investing more in immunity. How the sexes differ in their allocation to immunity and longevity when essential resources (e.g. protein) are scarce is not well studied.

Studies often test the effect of nutritional deficits on adult longevity by experimentally manipulating adult condition [36], [40]. However, the resources acquired during juvenile development not only have immediate effects on life-history and phenotype, but they can have profound long-term consequences in the adult [41]–[45]. Here, we manipulated the quality of the diet fed to Texas field crickets, *Gryllus texensis*, for their entire life to mimic an environment in which individuals experience a life-long shortage of nutritional resources. We then measured disease resistance of adult males and females using a host resistance test to determine how diet-restriction affects immune function and whether the effect is sex-specific. We also monitored the size and mass at eclosion of all individuals to test the prediction that individuals in better body condition (i.e. acquired more resources) allocate more resources to growth than individuals in poorer condition.

## Materials and Methods

Experimental crickets were lab-reared descendants of individuals collected in Austin, TX (USA) and were raised communally for their first three weeks in large bins (64 L) with water and dry cat food (Special Kitty: 34% protein, 13% fat) provided *ad libitum*. Crickets were then housed in separate individual containers (300 mL), provided with water and haphazardly assigned to either a low- (90% bran and 10% cat food) or high- (10% bran and 90% cat food) protein diet (n = 180 and 168, respectively) for the duration of the experiment. Experimental crickets were fed *ad libitum* and, food, water, and containers were replaced weekly. Crickets were reared and maintained at 27±1°C on a 12 h:12 h: light:dark cycle and were checked daily for eclosion to adulthood. At eclosion, the time to eclose (days), body mass (g), and pronotum length (mm), were recorded. Pronotum length (a proxy for body size) was defined as the distance between the anterior and posterior edges of the pronotum and was measured under a stereomicroscope using Leica LAS image analysis software (Leica Microsystems Inc., Buffalo Grove, IL, USA).

A host resistance test was used to determine an individual’s relative disease resistance (i.e. overall immune ability) [46]. To assess a cricket’s susceptibility to bacterial infection, we injected a LD50 dose (2.0×10^4^ cells/2 µl) of the bacterium *S. marcescens* into sexually mature (i.e. 7–11 days post-eclosion) virgin crickets using a microcapillary needle (each needle was used once only). *S. marcescens* is a Gram negative soil microbe commonly found in the cricket’s natural environment [21]. Crickets were cold anesthetized (4°C) prior to injection in the abdomen’s right side. Crickets were placed in a fresh cage and monitored for mortality every 12 hours for 5 days [21]. Crickets were given water and maintained on their experimental diet until death. Females could not oviposit at any time during the experiment.

**Figure 1:**
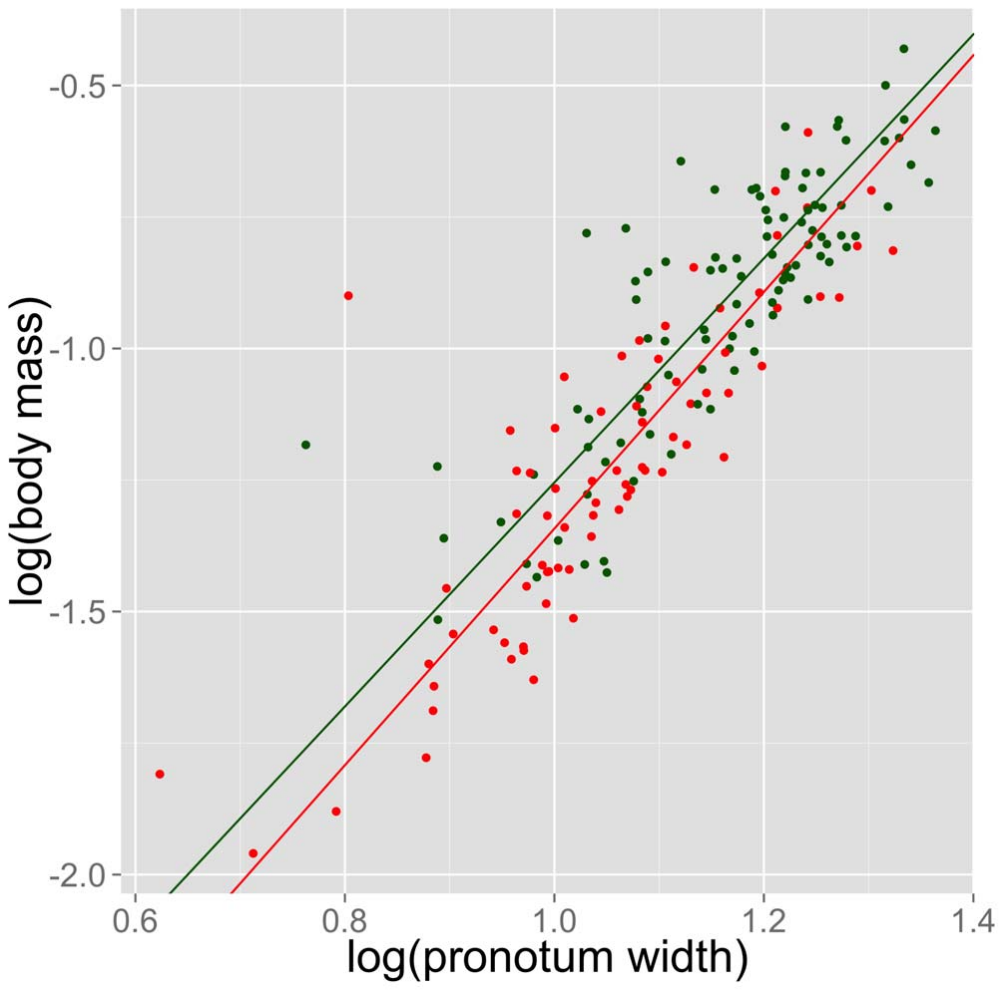
Model II regression slopes for low- and high-quality diet treatments. Reduced major axis regression slopes for low- (red) and high- (green) quality diet treatments did not significantly differ (likelihood ratio = 0.407, lamda = 4.76, p = 0.52; low-quality diet: slope = 2.248, slope 95% CI = 1.98–2.55, intercept = −3.951, n = 75; high-quality diet: slope = 2.129, slope 95% CI = 1.90–2.38, intercept = −3.384, n = 99).

Body condition at eclosion and at the time of bacterial injection was calculated for each individual using Peig and Green’s [47] scaling mass index (SMI). This index uses the equation SMI = M_i_[L_0_/L_i_]∧b_SMA_ to standardize individual mass (M_i_) to a specific fixed body size (L_o_ = mean size of data set; L_i_ = individual size) and is designed to incorporate allometric changes in scaling (b_SMA_) that are observed in many species. We first used model II regression to calculate the slope (b_SMA_) of the best-fit line from a standardized major axis regression of fresh body mass on pronotum length (both variables log-transformed). The scaling mass index is superior to other methods of determining body condition from mass and length estimates because its use of model II linear regression (i.e. standardized major axis regression) incorporates the likelihood that both variables have some underlying error rate associated with their measurement [48]. The model II slopes for the two diet treatments did not differ (likelihood ratio = 0.407, lambda = 4.76, p = 0.52; Figure 1), so we combined both treatment groups and used the common slope (b_SMA_ = 2.319) in our analysis. L_o_ is the mean pronotum length from the entire dataset (mean pronotum length = 3.073 mm). We calculated each individual’s SMI by substituting their fresh body mass (M_i_) and pronotum length (L_i_) into the equation SMI = M_i_[3.073/L_i_]∧2.319.

The effect of diet treatment and sex on the time required to eclose, and body mass, pronotum length, and body condition at eclosion were tested using multivariate ANOVA. Univariate ANOVAs were subsequently used to assess which dependent variables were affected by diet and sex. ANCOVA was used to test the effect of diet and sex on body condition (i.e. SMI values) at the time of the host resistance test while statistically controlling for post-eclosion age at injection. We used Cox regression (our data met the assumption of proportional hazards) to test the effect of diet and sex on survival after injection. Statistical analyses were conducted using the statistical environment R [49] within which model II regressions were conducted using *smatr* [50] and data were visualized using *ggplot2* [51].

## Results

**Figure 2:**
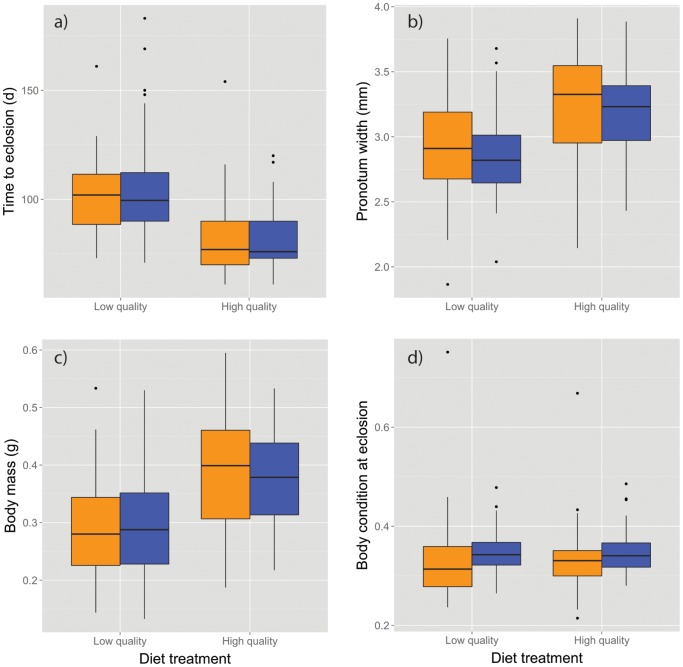
Phenotypic and life-history values for crickets fed low- and high-quality diets. Boxplots of a) development time, b) body size, c) body mass, and d) body condition for female (orange) and male (blue) crickets at the time of eclosion. The box represents the lower (25%) and upper (75%) quartiles, the solid dark horizontal line is the median, and the whiskers indicate 1.5 times the interquartile range. Data beyond the end of the whiskers are outliers and plotted as black points (low-quality females, n = 35; low-quality males, n = 40; high-quality females, n = 47; high-quality males, n = 52).

**Figure 3:**
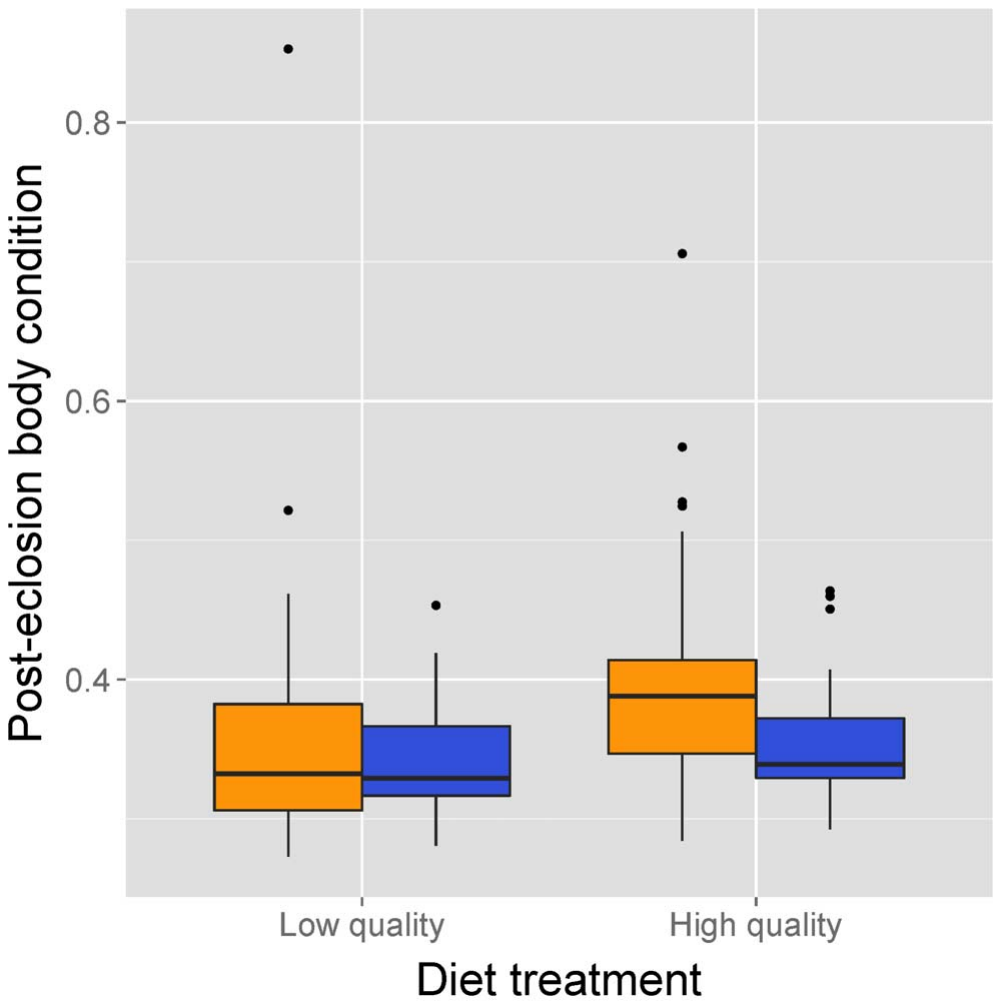
Sex-specific body condition of crickets fed low- and high-quality diets. Boxplots for the effect of diet and sex on body condition of crickets at the time of the host resistance test with live *Serratia marcescens*. Boxplot description and sample sizes are given in the caption for Figure 1.

A significantly greater proportion of crickets survived to eclosion on the high-quality diet (99/168 crickets) compared with those on the low-quality diet (75/180 crickets; *X* ^2^ = 9.68, df = 1, p = 0.001). MANOVA revealed a significant effect of diet treatment, but not sex, on the four life history traits measured for mature crickets (diet: Pillai’s trace = 0.392, F_4,167_ = 26.89, p<0.00001; sex: Pillai’s trace = 0.039, F_4,167_ = 1.674, p = 0.158; diet*sex interaction: Pillai’s trace = 0.012, F_4,167_ = 0.523, p = 0.72) (Figure 2). Univariate ANOVAs showed that crickets on the low-quality diet required significantly more time to eclose, and had significantly smaller mass and body size at eclosion than crickets on the high-quality diet (Figure 2; see summary statistics in Table S1). Although neither diet nor sex significantly affected body condition at eclosion, females were in significantly better condition at the time of the host resistance test (7–11 days post-eclosion) compared to males, (ANCOVA controlling for post-eclosion age, sex: F_1,169_ = 10.41, p = 0.002) and individuals fed a high-quality diet were in significantly better condition at the time of their host resistance test (diet: F_1,169_ = 3.94, p = 0.0488; diet×sex interaction: F_1,169_ = 2.20, p = 0.14; the covariate age at time of assay: F_1,169_ = 1.90, p = 0.17; Figure 3).

**Figure 4:**
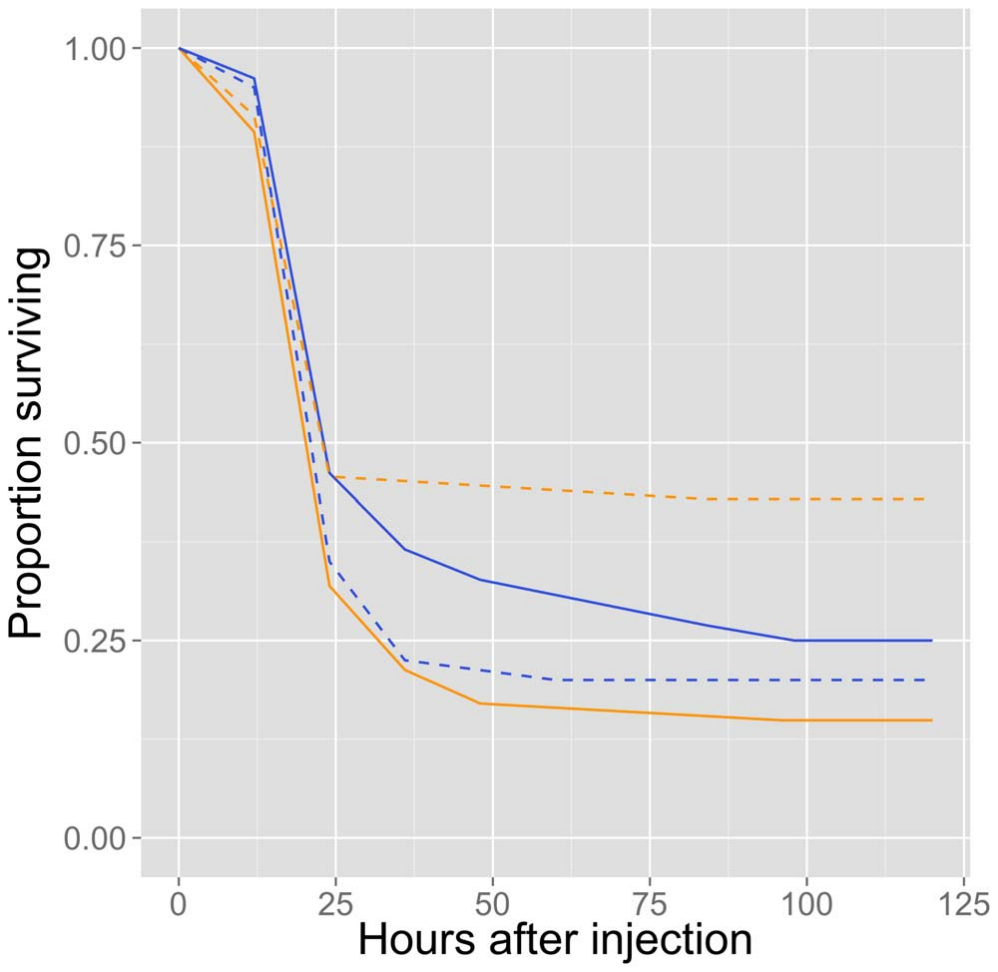
Sex-specific survival after infection of crickets fed low- and high-quality diets. Survival of adult females (orange lines) and males (blue lines) reared on a low- (dashed lines) and high-quality (solid lines) diet. Sample sizes are given in caption for Figure 1.

We found a significant diet*sex interaction in the adult survival analysis (Cox regression: z = 2.501, p = 0.0124, n = 174) so we proceeded to analyze the data for each sex separately. In a separate sex-specific analysis, we found no difference in post-infection survival between males reared on low- or high-quality diets (z = 0.804, p = 0.42, n = 92). In contrast, females reared on a low-quality diet had significantly greater survivorship after infection than those reared on a high-quality diet (z = 2.282, p = 0.0225, n = 82) (Figure 4).

## Discussion

The environment experienced by juvenile animals is expected to significantly affect adult life history decisions. Specifically, developing in a nutrient-poor environment should have detrimental effects on the adult phenotype and attendant life history traits because growth and self-maintenance are costly in terms of resource requirements. In line with this prediction, we found that *G. texensis* crickets on a low-quality diet required significantly more time to eclose, and eclosed at a smaller body size with lower mass than those on a high-quality diet. Our findings are congruent with those reported for other cricket species [42], [43], [52], [53] and confirm that our diet treatments successfully manipulated nutritional intake for each group of experimental crickets. Contrary to prediction, however, crickets reared on a high-quality diet were not in better body condition at the time of eclosion than those fed a low-quality diet. Perhaps because body condition is critical to a variety of fitness-related traits, individuals optimize development time to maximize condition at maturity [9].

Although there was no diet or sex effect on condition at eclosion, there was a difference 7–11 days afterward with females being in better condition than males and crickets on a high-quality diet being in better condition than those on a low-quality diet. Perhaps females in our study were in significantly better condition than males 7–11 days post-eclosion (at the time of injection with live bacteria) because they converted their dietary resources into body mass, most likely in the form of eggs and possibly body fat, with those fed a high-quality diet having more resources to convert. Judge et al. [43] also found that female *G. pennsylvannicus* fed a high-quality diet gained more mass 1–6 days post-eclosion, but the authors did not investigate the cause of this gain (i.e. whether ‘high-quality’ females had more eggs than ‘low-quality’ females). On the other hand, males in our study might have accrued less condition than females because they invested their resources into energetically costly sexual signals (e.g. courtship calling) [42]. That said, however, males on the high-quality diet in our study were apparently able to exceed the demand for resources used in calling and transform the surplus into body mass as these males significantly increased their condition 7–11 days post-eclosion. Hunt et al. [42] found that male calling was costly to condition as higher calling rates were correlated with a greater decrease in body mass. Contrary to our finding, neither Judge et al. [43] nor Hunt et al. [42] found that male crickets on a high-quality diet gained more mass post-eclosion than males on a low-quality diet.

Disease resistance in our study showed interactive effects of sex and diet. This is contrary to other studies showing that female animals generally have better immune function than males [12], [54], crickets included [21], [55]. By analyzing the sexes separately, however, we discovered a significant role of diet in disease resistance in one sex only and this difference was opposite to prediction. We predicted that crickets on a high-quality diet would have greater disease resistance because immune defence is generally found to be condition-dependent. Instead, we found that crickets maintained on a high-quality diet did not have significantly greater disease resistance than those on a low-quality diet. In fact, females fed a high-quality diet (and in better body condition) had significantly poorer disease resistance than females on a low-quality diet (and in poorer condition), whereas diet treatment had little effect on male survival. Our results therefore suggest that disease resistance in *G. texensis* might not be dependent on body condition at the time of infection.

Our contradictory finding is perhaps due to an adaptive allocation of resources to immunity at eclosion based on the perceived quality of the adult environment that was assessed during development. If survival is expected to be compromised after sexual maturity and reproductive success is contingent upon longevity, then individuals should invest more in processes, like immunity, that will improve survivorship in harsh environments. Some studies suggest that female fitness is more closely tied to longevity than it is in males 22,34, so it is possible that females in our experiment might have perceived a threat to their adult longevity, and hence reproductive success, and invested more in survival via increased disease resistance. The reproductive success of male *G. texensis*, on the other hand, might not be as tightly linked to longevity as in females, and so males might benefit from investing more in sexual signals (e.g. courtship calling) relative to immune defense.

Alternatively, McKean and Nunney [16], [56] argue that immunological sex differences are phenotypically plastic with environmental variation dictating the direction and magnitude of immunity. Instead of one sex possessing intrinsically superior immunity, they posit that perhaps sex differences in immune function are the result of sex-specific changes in reproductive behavior that arise due to variation in the availability of fitness-determining resources. For example, when male *D. melanogaster* experience greater availability of females, the fitness-determining resource, they increase their level of courtship, which apparently comes at the cost of decreased immune function [16], [56] and longevity [57]. In contrast, female fecundity in *D. melanogaster* is contingent upon food availability and so producing more eggs when food is available may improve female fitness more than by investing in prolonged longevity. Indeed, access to more food results in significant increases in egg production [56], but also in decreased longevity [58], [59]. In line with McKean and Nunney’s [56] hypothesis, the females in our study that were fed a high-quality diet experienced decreased disease resistance relative to poorly-fed females despite having superior body condition (body mass scaled to body size). That the well-fed adult females were in significantly better condition at the time of injection suggests that perhaps they invested more in egg production since eclosion than poorly-fed females. Further support for this hypothesis is seen in the lack of difference in disease resistance between diet treatments in adult males despite those on a high-quality diet being in significantly better condition. McKean and Nunney’s [56] hypothesis predicts that female, not food, availability should affect disease resistance in males.

At the mechanistic level, our observed elevated disease resistance in individuals fed poor diets could be due to increased expression of genes whose products fight pathogenic infections. Fellous and Lazarro [29] found that larval *Drosophila melanogaster* that were fed more protein exhibited greater constitutive transcription of two genes encoding defensive antimicrobial peptides as adults. Muturi et al. [24], however, found that starvation decreased the expression of immune-related genes in larval and adult *A. aegypti* mosquitoes and increased their susceptibility to viral infection.

The elevated expression of immune-related genes should manifest as increased immune function. Indeed, Brown et al. [60] found that diet-restricted *D. melanogaster* exhibit increased production of the antimicrobial agent nitric oxide. Diet restriction can also have sex-specific effects on immune effector activity. Klemola et al. [31], for example, showed that autumnal moths (*Epirrita autumnat*a) reared as larvae on low-quality diets had greater encapsulation ability than those on high-quality diets with the effect significantly stronger in females than in males.

Equally informative is that some studies have found no effect of juvenile diet on some immune responses in adult males, which may explain our results. For example, Jacot et al. [41] showed that the proPO cascade (involved in melanization and encapsulation of foreign bodies) in adult male *G. campestris* was unaffected by a restricted diet during development, and Simmons [52] found no effect of diet-restriction on lytic activity in male *Teleogryllus oceanicus*. In contrast, studies have also shown negative effects of a poor diet on immune effectors [41], [52], [61]. Given that diet restriction apparently affects immune parameters differently between the sexes and among taxa, we must be cautious when evaluating the effects of diet restriction on disease resistance when only a few effectors are assayed [46]. Moreover, given that the effect of diet restriction on disease resistance and tolerance is pathogen-specific, results must be evaluated on a pathogen-by-pathogen basis [61].

Increased immune function and disease resistance under dietary restriction might account, at least partially, for the observed increase in lifespan of animals [62], including crickets [63], [64], fed a restricted diet. Libert et al. [65], on the other hand, found that although dietary restriction increased expression of immune-related genes in *D. melanogaster*, reduced access to food did not significantly extend lifespan. Clearly, the interactions between sex, diet, immunity, and lifespan have complex effects on fitness and require considerably more experimental work to decipher.

## Supporting Information

Table S1 **Summary statistics (mean ±1 standard deviation) for effect of sex and diet on life history and phenotypic variables.**
(DOC)
