# Supplementary material for: Sex-Specific Effect of Juvenile Diet on Adult Disease Resistance in a Field Cricket
Source: PLoS One. 2013 Apr 18;8(4):e61301. doi: 10.1371/journal.pone.0061301 (PMC3630171; doi:10.1371/journal.pone.0061301)
Supplement: Table S1 — Summary statistics (mean ±1 standard deviation) for effect of sex and diet on life history and phenotypic variables. (DOC) [file pone.0061301.s001.doc]

| Table S1. Summary statistics (mean ± 1 standard deviation) for effect of sex and diet on life history and phenotypic variables. | | | | | |
| --- | --- | --- | --- | --- | --- |
|  | Low-quality diet | |  | High-quality diet | |
|  | Females  (n=35) | Males  (n=40) |  | Females  (n=47) | Males  (n=52) |
| Time to eclosion (days) | 100.97±18.50 | 105.50±23.93 |  | 82.34±18.12 | 81.98±12.95 |
| Body size at eclosion (mm) | 2.90±0.41 | 2.86±0.34 |  | 3.26±0.41 | 3.19±0.30 |
| Body mass at eclosion (g) | 0.29±0.09 | 0.30±0.09 |  | 0.38±0.10 | 0.38±0.07 |
| Body condition | 0.33±0.09 | 0.35±0.04 |  | 0.33±0.07 | 0.25±0.04 |
|  | | | | | |
